# A201 A CASE OF SYPHILIS MASQUARADING AS CROHN’S DISEASE

**DOI:** 10.1093/jcag/gwab049.200

**Published:** 2022-02-21

**Authors:** B C Smith, B Salh

**Affiliations:** 1 Internal Medicine, University of British Columbia, Vancouver, BC, Canada; 2 Vancouver General Hospital, Vancouver, BC, Canada

## Abstract

**Background:**

It is important for gastroenterologists to keep syphilis on their differential. We present a case of rectal syphilis initially misdiagnosed as Crohn’s disease with commensal intestinal spirochetosis.

**Aims:**

This case highlights how rectal syphilis can present with endoscopic and clinical findings consistent with refractory inflammatory bowel disease.

**Methods:**

Case report and literature review.

**Results:**

A 58 healthy male was referred for flexible sigmoidoscopy after having increased frequency of bowel movements, up to 10 per day, with mucous secretions at times mixed with blood and a 35lbs weight loss. He complained of urgency, tenesmus and intermittent incontinence. Endoscopy showed evidence of proctitis in the distal 10cm of the rectum with ulceration, edema and friability with biopsies showing moderately active colitis, compatible with ulcerative colitis.

5-ASA suppositories and subseqent oral prednisone had no effect. A colonoscopy 3 months later again showed proctitis and ulceration. Biopsies at this time showed moderately active proctitis with chronic architectural distortion and intestinal spirochetosis identified on Warthin-Starry stain in the right colon and rectum. Given the patient was immunocompetent he was diagnosed with inflammatory bowel disease and trialed on oral and rectal 5-ASA.

Four months later flexible sigmoidoscopy showed extensive distal rectal ulceration, biopsies positive for chronic proctitis and intestinal spirochetosis. Given no response to 5-ASA the patient was started on infliximab with a slight improvement but incomplete resolution of his symptoms. Repeat endoscopy showed continued rectal ulceration.

Incidentally, the patient had a same sex sexual encounter and subsequently tested positive for syphilis with positive *T. pallidum* EIA and an RPR of 1:8. He received benzathine penicillin G 2.4 million units intramuscularly.

After treatment with penicillin, the patient’s symptoms improved with no further hematochezia and decreased frequency. Flexible sigmoidoscopy showed normal mucosa with no evidence of ulceration indicating the initial proctitis and ulceration was secondary to infection by *T. pallidum.*

**Conclusions:**

Syphilis, known as the “Great imitator,” is on the rise in Canada, with cases of infectious syphilis increasing more than 259.5% over the past decade. Rectal syphilis is often misdiagnosed as it can present with variable symptoms including puritis, tenesmus, urgency of defecation and anal discharge that may be purulent, mucoid or bloody. Endoscopic findings include non-specific proctitis, masses, ulceration and pseudotumors. Diagnosis is made with serology as pathology cannot differentiate between commensal intestinal spirochetosis and *T. pallidum*.

Given the diversity of presentations and rise in incidence, gastroenterologists need to ensure they take a thorough sexual history and keep syphilis on their differential when encountering this common clinical scenario.

**Funding Agencies:**

None

